# Synergistic Dual Antibacterial Activity of Magnetite Hydrogels Doped with Silver

**DOI:** 10.1021/acs.langmuir.4c02964

**Published:** 2024-10-17

**Authors:** Mohamad Wehbe, Rayan Kadah El Habbal, Jad Kaj, Pierre Karam

**Affiliations:** Chemistry Department, American University of Beirut, P.O.Box 11-0236, Riad El-Solh, 1107 2020 Beirut, Lebanon

## Abstract

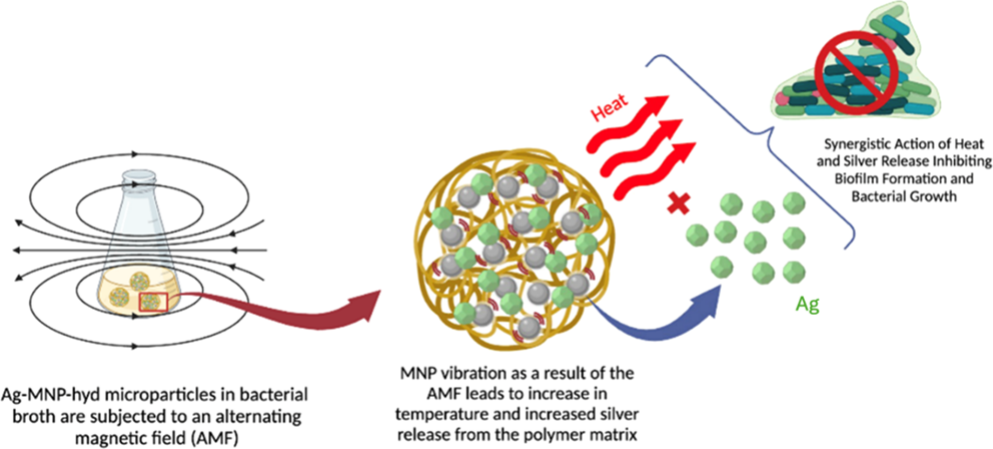

In this work, we utilized poly-*N*-isopropylacrylamide (NIPAM), magnetic nanoparticles (MNPs), and silver nitrate to prepare magnetic hydrogel microparticles doped with silver, which exhibited a dual antimicrobial effect. The antibacterial effect of these composites was mediated by the antimicrobial activity of silver and the magnetic hyperthermic induction, which we believe increased biofilm disruption and silver release into the surrounding bacterial biofilms. The prepared particles were characterized by using several analytical techniques. The particles exhibited a porous morphology impregnated evenly with silver nanoparticles, as observed by scanning electron microscopy (SEM). Furthermore, we examined the antibacterial activity of our microparticles against *Escherichia coli* by determining the minimum inhibitory concentration (MIC) and minimum bactericidal concentration (MBC). Our findings revealed that the composites demonstrated significant antibacterial activity of up to 81% under magnetic hyperthermia as compared to 45% when samples were heated to the same temperature in a water bath at constant silver concentration. This demonstrates the distinctive inhibitory features of MNPs in enhancing bacterial killing when a magnetic field is applied. The findings of this study lay the groundwork for further exploration of microparticle-based antimicrobial therapies, which can contribute to the development of more advanced wound healing devices and better sterilization methods for medical devices.

## Introduction

Historically, infectious diseases have always been a major cause of mortality. During the early 20th century, infectious diseases were a public healthcare burden, where the three leading causes of death were pneumonia, tuberculosis, and diarrhea, contributing to a third of all deaths worldwide.^1^ Developments in medicine, sterilization, and prevention techniques helped greatly in reducing the burden associated with these infectious diseases where now the leading cause of death is heart disease.^1^ The rise of antibiotics with the discovery of penicillin led to the start of the golden age of antibiotic development, leading to a sharp decline in mortality and a 29-year increase in life expectancy.^2,3^ By the 1960s, multiple classes of antibiotics were developed, leading many to believe that infectious diseases have been conquered for good. However, starting in the early 1990s, there has been an alarming resurgence of new infectious diseases that could no longer be treated using older antibiotics. Moreover, despite our advances in sterilization, hygiene, and prevention of infectious spread, infectious diseases remain one of the top causes of death globally especially in middle- and low-income countries.^4^ With the ability to constantly evolve, microbes have become resistant to many of the currently described antibiotics we have. Therefore, bacterial infections resistant to multidrug regimens have become a global concern. This surge in antimicrobial resistance has elicited a global healthcare alarm, underscoring the need for new antimicrobial therapies.

In response, there has been significant exploration and development of alternative antimicrobial materials, including natural extracts with antimicrobial properties and heavy metals like silver. Recently, silver has gained a lot of attention because of its ability to be used as an alternative or adjunct to antibiotics.^5−11^ This has prompted extensive research to elucidate the different mechanisms of silver’s antimicrobial activity. However, a finite mechanism of silver’s action on bacterial cells has yet to be determined with many studies describing different potential mechanisms of action of antibacterial activity.^12−14^ Because of its size and shape and the surface area to volume ratio, it has been proposed that silver nanoparticles (AgNPs) attach to the bacterial surface making their membranes more permeable and prone to dissolution.^15,16^ While AgNPs have been used for their antimicrobial effect, it is the release of silver ions from these nanoparticles that primarily contributes to its reported antimicrobial effect.^17^ The increase in cell’s permeability allows AgNPs (20–50 nm) to penetrate the microbe, damaging its mitochondria and ribosomes as well as disrupting DNA and generating free radicals that are toxic to cells.^17−20^ Previously, our lab has developed multiple composites that inhibit antibacterial activity through different mechanisms. Preparation of antimicrobial composites such as microsized cross-linked polyvinylpyrrolidone decorated with AgNPs or silver postmetalated-zirconium metal organic framework (MOF)-based crystals have all inhibited growth of *Escherichia coli* through the mechanism of silver release into solution.^21,22^ Our group has also developed composite membranes of polyvinyl chloride loaded with MOFs postmetalated with silver, showing antibacterial potency by a contact-based inhibition mechanism.^23^ We demonstrated that AgNPs effectively inhibit bacterial growth by disrupting biofilm formation through contact-based inhibition. Numerous studies have extensively highlighted silver’s strong inhibitory effect on biofilm formation in both Gram-positive and Gram-negative bacteria.^11,24−30^ Moreover, despite their effectiveness as antimicrobial agents, these materials still present challenges such as lack of controlled release, high concentration requirements, limited bioavailability, and potential toxicity.^31^

In addition to the extensive types of nanoparticles that could potentially be used in antibacterial therapies, magnetic nanoparticles particles (MNPs) have been regarded as an excellent supporting material in antibacterial applications.^32,33^ MNPs are unique in their ability to generate heat when an alternating magnetic field is applied. It has been shown that the hyperthermia generated by these MNPs exhibited a synergistic effect when combined with antibiotics.^25^ This is because the increase in temperature by 5 °C or more results in the dispersal of bacterial biofilms leading to better antibiotic penetration and thus better antibacterial activity.^34^

Hydrogels, a class of versatile polymeric biomaterials, have emerged as promising candidates for antimicrobial delivery due to their ability to provide prolonged and controlled release of therapeutic agents preventing their cytotoxic effects.^35−38^ Because of their ability to mimic properties of the body with their porous structure, high water content, and high fluid absorptive capacity, hydrogels have become a staple in tissue engineering.^39,40^ However, hydrogels also face limitations, such as mechanical instability, reduced drug loading capacity, and difficulty maintaining the bioactivity of loaded agents, especially when derived from natural polymers. Synthetic hydrogels, while more stable, can suffer from network defects that impact the bioactivity and diffusion rates of the incorporated drugs.^41−43^

Herein, this work aims to develop a new prototype for dual bacterial inhibition by combining the hyperthermic properties of MNPs with the antibacterial properties of silver nanoparticles. MNPs and *N*-isopropylacrylamide (NIPAM) were combined to form magnetic poly-NIPAM microparticles (MNP-hyd), and then silver nanoparticles were reduced on their surface to form a matrix of MNP hydrogels with silver (Ag-MNP-hyd). As proof of concept, the antibacterial activity of these microparticles was evaluated against *E. coli* under 3 different conditions: no heat, hyperthermia, and regular heating. The antibacterial effects of these microparticles were then evaluated by describing their MIC, MBC, and bacterial concentrations in colony forming units (CFUs). Our Ag-MNP-hyd composites represent a significant advancement, offering control, stability, and efficiency through a synergistic combination of heat and silver for antibacterial therapy.

## Materials and Methods

### Materials

*N*-isopropylacrylamide (NIPAM) (97%), *N*,*N*′-methylenebis(acrylamide) (BIS) (99%), iron(III) chloride hexahydrate (97%), anhydrous iron(II) chloride (98%), sodium citrate dihydrate (≥99%), l(+)-ascorbic acid (99%), ammonium hydroxide (28–30%), and Luria–Bertani (LB) Broth were purchased from Sigma-Aldrich. Potassium persulfate (KPS) (≥99%), silver nitrate (≥99%), nitric acid (65%), and Mueller Hinton Broth (MHB) were purchased from Fisher Scientific. Phosphate Buffer Saline (PBS) was purchased from Lonza Bioscience. 18.2 MΩ-cm deionized water was used in all experiments.

### Methods

#### Bare Hydrogel Synthesis

Bare hydrogels were synthesized using a slightly modified method by adding NIPAM (71 mM), BIS (5.1% mol %), and KPS (3.1 mM) into a round-bottom flask while maintaining a nitrogen-purged environment. The reaction mixture was sealed and heated in an oil bath at 60 °C for 12 h under continuous stirring at 300–400 rpm.^44^

#### Citrate-Coated Iron Oxide Nanoparticle Synthesis

A slightly modified coprecipitation method was utilized for the synthesis of citrate-coated iron oxide nanoparticles (MNPs).^45^ In brief, a mixture of FeCl_3_·6H_2_O and anhydrous FeCl_2_ was mechanically stirred in deionized water under a nitrogen atmosphere to achieve respective concentrations of 0.15 and 0.075 M. Subsequently, a 1.6 M solution of ammonium hydroxide (28–30%) was introduced dropwise. The reaction was allowed to stir for 30 min before the addition of sodium citrate dihydrate at a final concentration of 0.17 M. The mixture was then heated to 90 °C using a water bath and left for 45 min to get rid of excess ammonium hydroxide. The nanoparticles were separated from the solution by magnetic decantation and were thoroughly purified using ethanol and acetone. Specifically, larger MNPs that settled at the bottom were discarded, while the smaller, nonprecipitating MNPs remaining in the supernatant were then used. This was followed by drying the MNPs for 12 h. Finally, they were dispersed in deionized water. Centrifugation at 4500 rpm for 15 min was performed to get rid of larger particles.

#### MNP-Hydrogel (MNP-hyd) Synthesis

**Scheme 1:**
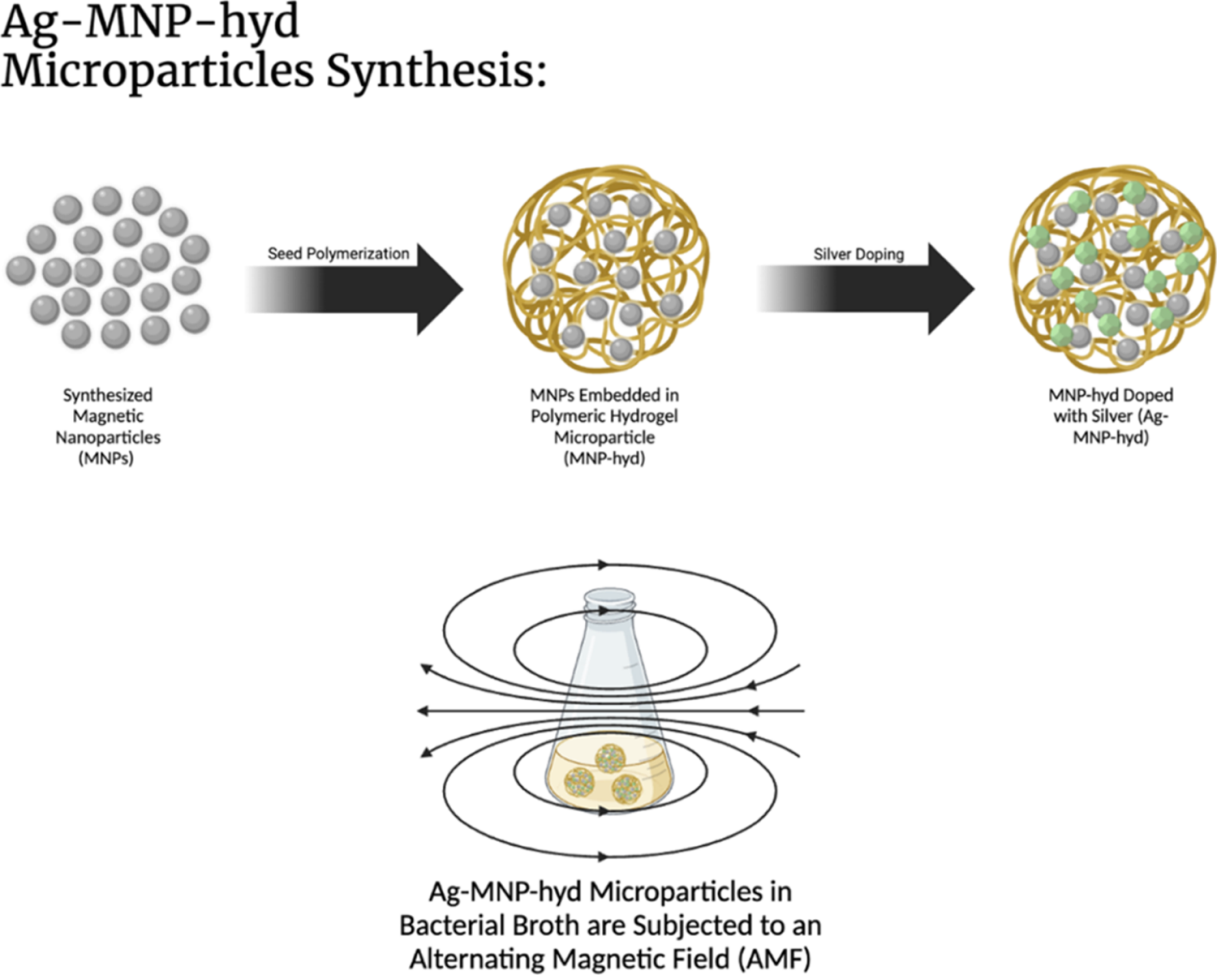
Schematic Representation of the Synthesis of Ag-MNP-hyd and Its Application in the Presence of an AMF The Ag-MNP-hyd particles are shown suspended in a bacterial broth, illustrating the process and interaction within the medium.

We followed a seed polymerization method for the synthesis of the MNP-doped pNIPAM hydrogels (MNP-hyd)^46^ and modified it to physically incorporate our MNPs (Scheme 1). In a round-bottom flask containing 50 mL of MNPs at a concentration of 30 mg/mL, NIPAM (71 mM), BIS (5.1% by mol), and KPS (3.1 mM) were added, while keeping the solution purged with nitrogen. The reaction flask was then sealed and heated for 12 h at 60 °C using an oil bath, all while stirring at 300–400 rpm. The particles were separated and washed with DI water twice by virtue of centrifugation at 19,000 rpm for 20 min, at each step. The resulting MNP-hyd were freeze-dried for 12 h to obtain a dry aerogel. It is important to mention that Initially, we used a concentration of 15 mg/mL MNPs; however, this concentration was insufficient to achieve the desired heating response under the application of an alternating magnetic field, reaching only 30 °C. To address this, we increased the MNP concentration to 30 mg/mL, which allowed us to achieve the target heating temperatures. We observed that concentrations of >30 mg/mL adversely affected the colloidal stability of the microparticles, leading to structural and functional instability. Therefore, the 30 mg/mL concentration was identified as the optimal balance, providing effective heating while maintaining the colloidal stability of the MNP-hyd microparticles.

#### Silver-MNP-Hydrogel (Ag-MNP-hyd) Synthesis

To generate silver diamine, a solution of 35 mM AgNO_3_ in 5 M NH_4_OH (7.5 mL in total) was first prepared. It was then added to 10 mL of a 10 mg/mL MNP-hyd dispersion, followed by the dropwise addition of 5 mL of 1.14 M ascorbic acid (15–20 min). All steps were done while stirring. The resulting mixture was washed and dried following the same steps as those with MNP-hyd above.

#### Heating/Induction Experiments

A total of nine vials were prepared and divided into three sets. Each of the bare hydrogels (hyd), MNP-hyd, and Ag-MNP-hyd was weighed into one vial of each set, giving rise to three replicates of each material. All were weighed to the same mass and dispersed in 1 mL of broth by vortexing. The broth contained bacteria that were grown and diluted to a suitable concentration prior to this experiment. Set 1 was used as a control and, therefore, was not heated. The temperature for each vial was measured right before starting with heating. One set of three vials (hyd, MNP-hyd, and Ag-MNP-hyd) was heated using a water bath (conductive heating), and the other was heated using an alternating magnetic field AMF (inductive heating using NanoTherics MagneTherm with a built-in-coil NAN201003 at a 109.5 kHz frequency with a voltage set between 30 and 32 V). To prevent precipitation during heating, we implemented a protocol where the dispersions were mixed at regular intervals of every 2 to 5 min. Heating was done for 15–20 min and the heating temperature was determined per batch, usually between 43–50 °C, as determined by the samples being inductively heated. After the vial was heated, the temperature of each vial was taken again before proceeding with bacterial quantification.

#### Antibacterial Properties Tests

The antibacterial properties of Ag-MNP-hyd were tested against Gram-negative *E. coli* (ATCC 25922). The minimum bactericidal concentration (MBC), which describes the concentration of silver needed to kill bacteria, was calculated according to The Clinical & Laboratory Standards Institute (CLSI) protocols and as described in more detail by Wiegand et al.^47^ First, 2–3 bacterial colonies were scraped from the surface of a freshly prepared plate and inoculated into 3 mL of LB broth, which was then set into an incubator shaker at 37 °C and 200 rpm. The overnight culture was then centrifuged, and the supernatant was discarded. The formed pellet was then vortexed with 5 mL of PBS solution. 100 μL of the obtained suspension was then added to 3 mL of MHB and set in the incubator shaker for 4 h at 37 °C and 200 rpm. The absorbance of the obtained bacterial growth was then measured using a NanoDrop 2000c spectrophotometer (Thermo Fisher Scientific) and subsequently diluted to be in the range of the 0.5 McFarland standard (OD_600_ nm between 0.08 and 0.013). The final suspension was then diluted to reach a bacterial concentration of 1 × 10^5^ and 1 × 10^6^ CFU/mL.

Different concentrations of hyd, MNP-hyd, and Ag-MNP-hyd were then weighed and then sterilized under ultraviolet (UV) light for 20 min. These composites were each suspended in 1 mL of the final bacterial suspension. After heating and induction of the different composites, these solutions were then incubated at 37 °C and 200 rpm for 24 h. The turbidity of the samples could not be assessed visually because of the suspended composites. Consequently, these composites were then collected by a magnet, and the absorbance of the suspension was then assessed using a NanoDrop 2000c spectrophotometer. After determining the absorbance of each sample, serial dilutions were performed for each sample to obtain three different concentrations of each: 1/10, 1/100, and 1/1000. 100 μL of each sample were then plated on LB agar plates to determine the bacterial concentration. As for the MBC, defined as the lowest concentration of silver that resulted in 99.9% bacterial killing, it was determined by taking 100 μL aliquots from the original growth tubes and plating them. All LB agar plates were then incubated at 37 °C for 24 h and then visually inspected for colony growth. To calculate the CFU/mL of the original sample, the number of CFUs on the countable plate was multiplied by 1 over the final dilution factor, which considers all of the dilutions of the original sample. Percentage bacterial inhibition was calculated using the following formula:


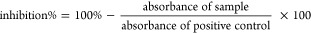


#### Material Characterization (SEM, XRD, TGA, DLS, AAS)

Our bare hydrogels MNP-hyd and Ag-MNP-hyd were imaged on a SEM (TESCAN MIRA3 LMU) under vacuum to determine their approximate size and surface morphology (SE detector used). Surface composition was also evaluated using energy-dispersive X-ray spectroscopy (SEM/EDX) with an OXFORD EDX detector. The material was deposited on a carbon tape and coated with either 15 nm gold or platinum. The beam intensity was set between 15 and 20 kV, depending on the material.

MNP-hyd and Ag-MNP-hyd were also characterized by using powder X-ray diffraction (PXRD) to assess their crystallographic structures and phase compositions. PXRD patterns were collected by using a Bruker D8 advance X-ray diffractometer (Bruker AXS GmbH, Karlsruhe, Germany) at 40 kV and 40 mA (1600 W) using Cu Kα radiation (*k* = 1.5418 Å).

To determine the hydrodynamic size of our microparticles, we employed dynamic light scattering (DLS). Our hyd, MNP-hyd, and Ag-MNP-hyd composites were dispersed in DI water and measured (average of three measurements for each) on a Particulate Systems NanoPlus zeta/nanoparticle analyzer.

To quantify the amount of silver in our samples and test the thermal stability of our particles, unlike SEM/EDX that was used to qualitatively visualize and detect surface silver, we utilized two different techniques/instruments. The first utilized thermogravimetric analysis (TGA) using a NETZSCH TG 209 F1 Libra. Briefly, around 15 mg of the hyd, MNP-hyd, and Ag-MNP-hyd were weighed into different Al_2_O_3_ crucibles and loaded into the TGA. They were then steadily heated at a rate of 20 °C/min until a final temperature of 1100 °C while monitoring the change in the mass of the samples. Thus, we can calculate the % weight attributable to silver in our particles, assuming that no MNPs were lost between MNP-hyd and Ag-MNP-hyd, as follows: 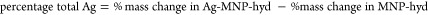
The other technique utilized to determine total silver is through acid digestion of a Ag-MNP-hyd sample and quantification using flame atomic absorption spectroscopy (flame-AAS). Briefly, around 10 mg of the sample was digested in hot HNO_3_ (65%) for 10–15 min, filtered using 0.22 μm syringe filters, and diluted to different extents. Standard solutions of AgNO_3_ were prepared by pipetting from a stock solution of 100 mg/mL AgNO_3_. All dilutions and preparations were made in 0.15 M HNO_3_. The standard solutions and diluted Ag-MNP-hyd samples’ absorption was then measured (at 328.1 nm) on a Thermo Fisher Scientific iCE 3000 Series AAS (flame mode) with a silver hollow cathode lamp. Flame type was a combination of acetylene and air (1:1) at an acetylene flow of 1.1 L/min, and background correction was also applied using a Deuterium lamp. The total amount of silver here will be as follows:
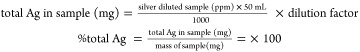
AAS was also used to quantify the amount of silver released by the Ag-MNP-hyd with and without heating (both conductively and inductively). Briefly, three vials containing an equal mass of Ag-MNP-hyd were prepared in DI water. One was not heated; the other was heated in a water bath; and the last was heated inductively. After heating for 20 min, all three samples were centrifuged at 13,500 rpm. The supernatant was then microfiltered and diluted to 5 mL with 0.15 M HNO_3_. Their absorptions were then measured. The same standard solutions were used as described previously.

## Results and Discussion

### Preparation and Properties of the Hybrid Microparticles

MNPs were synthesized using a coprecipitation method which involves mixing FeCl_3_·6H_2_O and anhydrous FeCl_2_ in deionized water under a nitrogen atmosphere followed by dropwise addition of ammonium hydroxide. After the addition of sodium citrate dihydrate followed by heating the mixture to 90 °C, MNPs were then decanted and purified and finally dispersed in deionized water. The size of our synthesized MNPs was then measured by using DLS, resulting in an average hydrodynamic radius of 40.5 nm (Table 1).

**Table 1:** Average Size of the Different Particles Synthesized Measured by DLS

material type	bare MNP	bare hyd	MNP-hyd	Ag-MNP-hyd
mode size (nm)	40.5	1199	1066	2265

**Figure 1:**
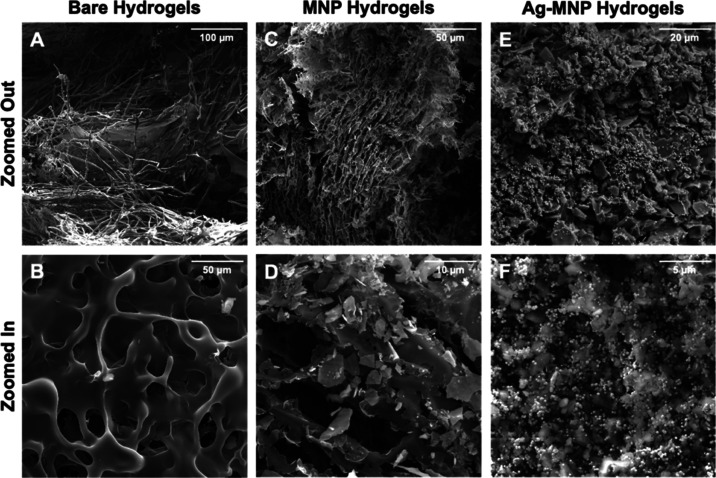
SEM images comparing the morphology of bare hydrogels (hyd), MNP hydrogels (MNP-hyd), and Ag-MNP hydrogels (Ag-MNP-hyd at 40 mg of silver).

**Figure 2:**
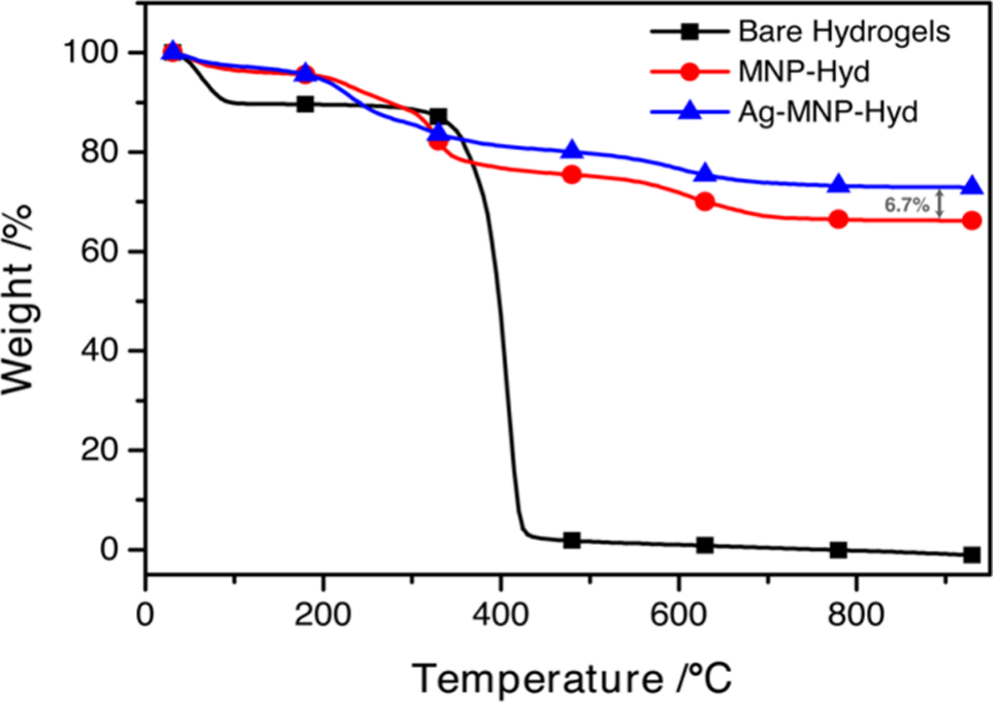
Thermal gravimetric analysis (TGA) of hydrogels (hyd), magnetic nanoparticle hydrogels (MNP-hyd), and MNP-hyd doped with silver (Ag-MNP-hyd).

Next, to synthesize our hybrid MNP-hyd microparticles, we followed a seed polymerization method whereby a solution of MNPs was mixed under nitrogen with specific concentrations of NIPAM, BIS, and KPS followed by heating at 60 °C in an oil bath for 12 h. After separating and washing the resultant solution, we obtained MNP-hyd microparticles of an average hydrodynamic radius of 1066 nm, which shows an increase in the average size of our particles compared to bare MNPs and are similar in size to bare hydrogels (Table 1). SEM images of MNP-hyd microparticles show a polymer-like configuration, confirming that the polymerization process was successful (Figure 1). TGA of MNP-hyd microparticles showed that the weight % remaining was higher compared to bare hydrogels. This is due to the presence of MNPs in the sample (Figure 2).

To synthesize our final Ag-MNP-hyd hybrid microparticles, we prepared a solution of silver diamine, which was then combined with an MNP-hyd dispersion, followed by the dropwise addition of ascorbic acid. The obtained solution was then washed and dried to yield our final hybrid microparticles doped with silver. DLS studies on our obtained microparticles yielded an average size of 2265 nm which was greater than that of MNP-hyd (Table 1 and Figure S1). This shows that the doping of silver on MNP-hyd silver yielded an increase in its average size. SEM images of Ag-MNP-hyd particles showed numerous silver nanoparticles scattered uniformly all throughout the MNP-hyd polymer surface (Figure 1). TGA of these hybrid microparticles showed that the weight % remaining was 6.7% higher than that of MNP-hyd (Figure 2). This 6.7% increase is most likely attributed to the additional silver nanoparticles doped on the surface of these microparticles. Atomic Absorption Spectroscopy analysis of Ag-MNP-hyd dispersed in deionized water yielded an average silver release of 9.83 × 10^–2^ μg/mL. AAS will be utilized later to assess the effect of heating by a water bath and by induction on overall silver release.

Finally, the XRD patterns of MNP-hyd and Ag-MNP-hyd are shown in Figure S2 and both exhibit characteristic peaks at approximately 30, 35, 43, 57, and 63° 2θ, which correspond to the (220), (311), (400), (511), and (440) planes, respectively, of the cubic structure of magnetite (JCPDS number # 19–0629). The peak broadening observed is consistent with the small size of the synthesized nanoparticles. It is noteworthy that no characteristic peaks of crystalline Ag species could be clearly observed. This is probably due to the low silver content in the sample.

### Determining the Appropriate Silver Concentration

When doping MNP-hyd composites with silver, we tested different weights of AgNO_3_ for the synthesis of Ag-MNP-hyd to determine the optimal amount of silver. We first started by using 180 mg of AgNO_3_ by using the doping steps detailed above. We then tested the growth inhibition and bactericidal effect of our prepared composites against *E. coli* at different concentrations of the prepared Ag-MNP-hyd. The percentage inhibition of *E. coli* was calculated relative to a positive control of 100% bacterial growth in the absence of any composites after 24 h of incubation. These composites exhibited 95% bacterial inhibition with an MIC of 1.63 mg/mL while the MBC was determined at 3.3 mg/mL reaching an inhibition of 97%. As impressive as these initial results may seem, the silver concentration needed to be decreased. First, SEM images of our particles show clear stacking and overcrowding of silver on their surfaces (Figure S3). Second, subjecting these composites to a magnetic field barely increased the temperature. For example, increasing the Ag-MNP-hyd concentration to 25 mg/mL, which is 8 times greater than what was needed to reach MBC, only caused an increase in temperature from 21 to 31 °C after 20 min of induction.

The temperatures reached by our initial microparticles were not high enough to lead to an enhancement in antibacterial activity because the bacterial activity is not impacted by temperatures below 38 °C.^48^ Surface silver overcrowding, as mentioned previously, raises concerns about structural instability and uncontrolled sporadic release of silver into the solution.^49−51^ When subjecting 25 mg/mL MNP-hyd to similar heating conditions by induction, temperatures increased from 21 to around 40 °C providing us with an insight that greater silver concentrations compromise heating by induction. The mechanism of this observation will be the subject of future investigations.

Because of the larger surface area and aggregate size of the silver nanoparticles, as viewed in Figure S3, we hypothesize that these interactions decrease the hyperthermic efficiency of our particles and thus lead to slower heating conduction from the microparticle matrix to the bulk solution. Thus, in favor of obtaining a more stable and controlled release of silver and better heating by induction, we decreased the silver concentration to a degree where it would allow appropriate heating by induction to a temperature of at least 45 °C as well as have enough silver particles to kill bacteria. In our optimization process, we then tested the synthesis of Ag-MNP-hyd using 80 mg of AgNO_3_ and 40 mg. We then tested the heating of each Ag-MNP-hyd under AMF for 15 min and measured the temperature before and after induction (Figure S4). Ag-MNP-hyd synthesized with 40 mg of AgNO_3_ showed the best heating under AMF (Figure S4). This allowed us to better elicit the dual synergistic antibacterial activity of silver with the inductive heating of MNPs, both integrated into our Ag-MNP-hyd microparticles.

**Figure 3:**
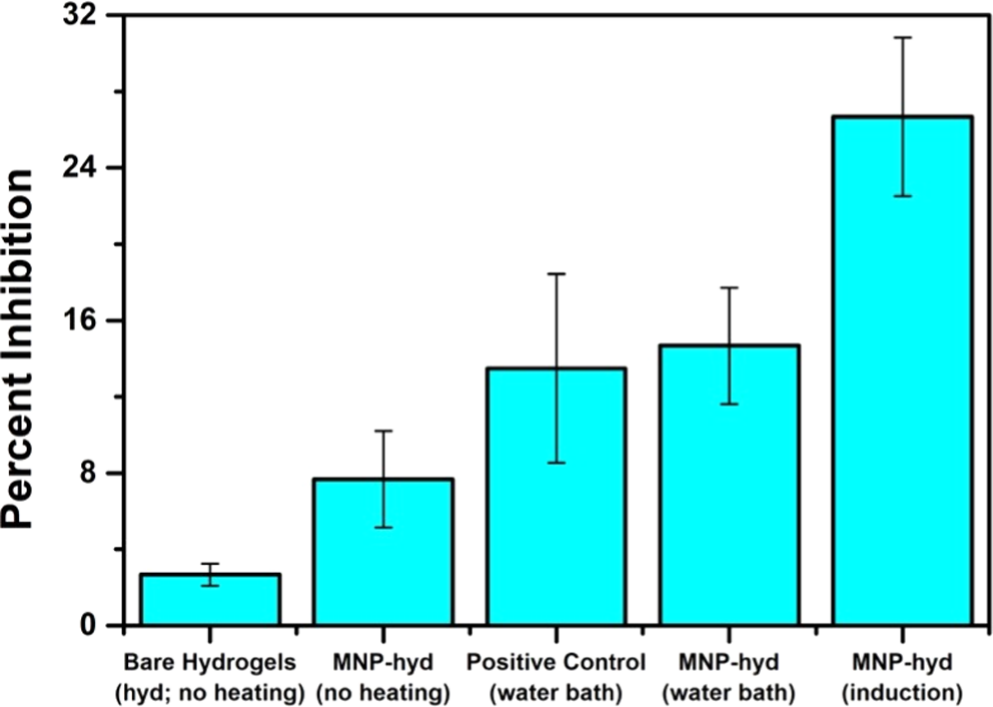
Relationship between bacterial inhibition (%) and different nonmetalated microparticles (25–40 mg/mL).

### Synergistic Antimicrobial Effect of Ag-MNP-Hyd

Using the same doping steps described previously, we decreased the mass of AgNO_3_ to 40 mg. SEM images show clear spacing and uniformity of silver nanoparticles spread out and embedded evenly on the magnetite-hydrogel matrix’s surface (Figure 1), which contrasts with the stacked and overcrowded nature of silver nanoparticles’ surface coverage in the case of 180 mg of AgNO_3_ (Figure S3). EDX/SEM data confirm that the nanoparticles (NPs) we see deposited on the surface of the Ag-MNP-hyd samples in Figures 1E,F are silver (Figures 1 and S5). The surface of our microparticles was coated with silver in a controlled manner, as shown in the SEM images (Figure 1E,F).

Furthermore, TGA of our composites gives us a good estimate of the potential overall silver by weight % present in our final synthesized composites (Figure 2). It is also important to note that the thermal stability of MNPs was not impacted by the introduction of silver onto the surface, as the slight difference in weight percentage seen is attributable to the presence of silver.

First, and as a proof of concept, we tested the growth inhibition and bactericidal effect of MNP-hyd composites against *E. coli* at 45 °C by both induction and a water bath. The concentration of the composites in each vial was at a concentration between 25 and 40 mg/mL in such a way that it maintained the mentioned temperature range (Figure S6). This had to be done to account for the slight variations in MNP doping between batches. The percentage inhibition of *E. coli* was calculated relative to a positive control of 100% bacterial growth in the absence of any composites after 18 h of incubation. *E. coli* incubated with bare hydrogel microparticles exhibits an average inhibition of 3% (Figure 3). On the other hand, incubating *E. coli* with MNP-hyd of the same concentration with no heating showed an average inhibition of 8% (Figure 3). This bacterial inhibition of magnetic nanoparticles has been reported previously in the literature. It has been shown that Fe_3_O_4_ nanoparticles exhibit an antibacterial effect owing to the generation of reactive oxygen species, which include superoxide, hydroxy, and singlet oxygen radicals, which penetrate bacterial cells, damaging their proteins and DNA.^52^ When subjecting MNP-hyd to heating by water bath or induction at 45 °C, bacterial inhibition increased. Heating using a water bath led to a 15% bacterial inhibition, 7% more than without any heating, which can be simply contributed to more stresses on the bacteria, making it more susceptible to the action of MNPs.^53,54^ Similarly, heating the bacterial broth without microparticles (positive control) at 45 °C for 15 min resulted in 14% inhibition of bacterial growth (Figure 3). These findings indicate that heating with a water bath, whether in the presence or absence of MNP-hyd, produces similar inhibition responses. This suggests that MNP-hyd alone exhibits negligible or minimal antimicrobial activity against *E. coli*, and most of the observed inhibition can be attributed to the heating effect. Heating by an alternating magnetic field (AMF) to the same temperature led, however, to a more significant inhibition of 27% (Figure 3). This significance in antibacterial activity is even more pronounced when the inhibition of the composites is normalized (Figure S7). When MNPs are subjected to a magnetic field, they absorb energy from the alternating magnetic field and efficiently transmit it in a highly localized form of heat. Kim et al. were successful in selectively killing *Staphylococcus aureus* bacteria without having tissue injury by subjecting the MNP-bound microorganisms to a high amplitude magnetic field.^55^ The increased inhibition seen when subjecting the bacterial cells to heating by magnetic induction can be also due to the disruption of bacterial biofilms formation. Addressing bacterial biofilms is important as their formation leads to a 10- to 1000-fold increase in antibacterial resistance.^56^ A recent paper highlights the synergistic effect of inductive heating and antibiotic action in killing and disrupting biofilm formation of *S. aureus* on metal joint implants.^57^ Some studies have reported that MNP biofilm disruption happens mechanically. Baig et al., for example, showed that Ag–Fe_3_O_4_@MoS_2_ MNPs create literal microchannels as they mechanically dig their way through the biofilm when subjected to an AMF.^58^ It was also observed that in hyperthermia, heating the suspension by MNPs to temperatures close to 43 °C resulted in bacterial death.^59^ MNPs are believed to heat their microenvironments at more elevated temperatures than that of the bulk solution,^60,61^ which highlights that MNP-induced hyperthermia by induction might be more efficient in inhibiting bacterial growth due to localized heating. In other words, when our bulk solution temperature was 45 °C, it is believed that the temperature near the MNP-matrix, comparable to the scale of a microorganism, is significantly higher. All of these aforementioned points explain why MNP-hyd mediated heating to 45 °C by induction showed enhanced bacterial inhibition compared to MNP-hyd composites heated through conduction/convection. Given these observations, quantifying these microenvironmental temperatures could help us better understand how localized heating contributes to the antibacterial efficacy of MNPs, potentially guiding the optimization of such systems in the future.

**Figure 4:**
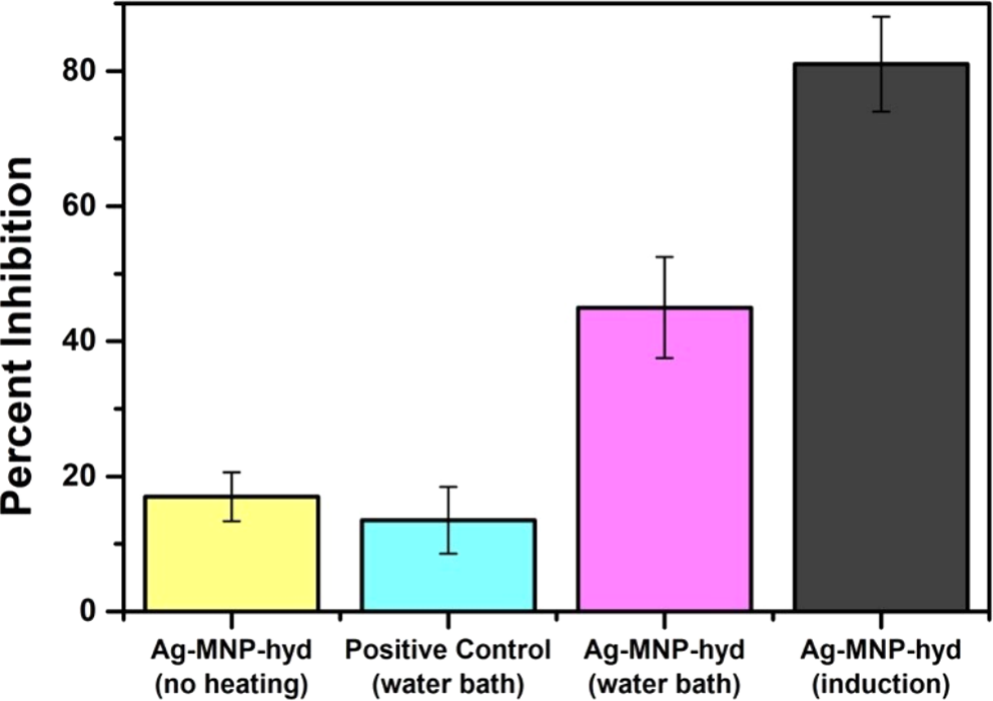
Relationship between bacterial inhibition (%) and postmetalated microparticles (40 mg/mL) under different heating conditions.

**Figure 5:**
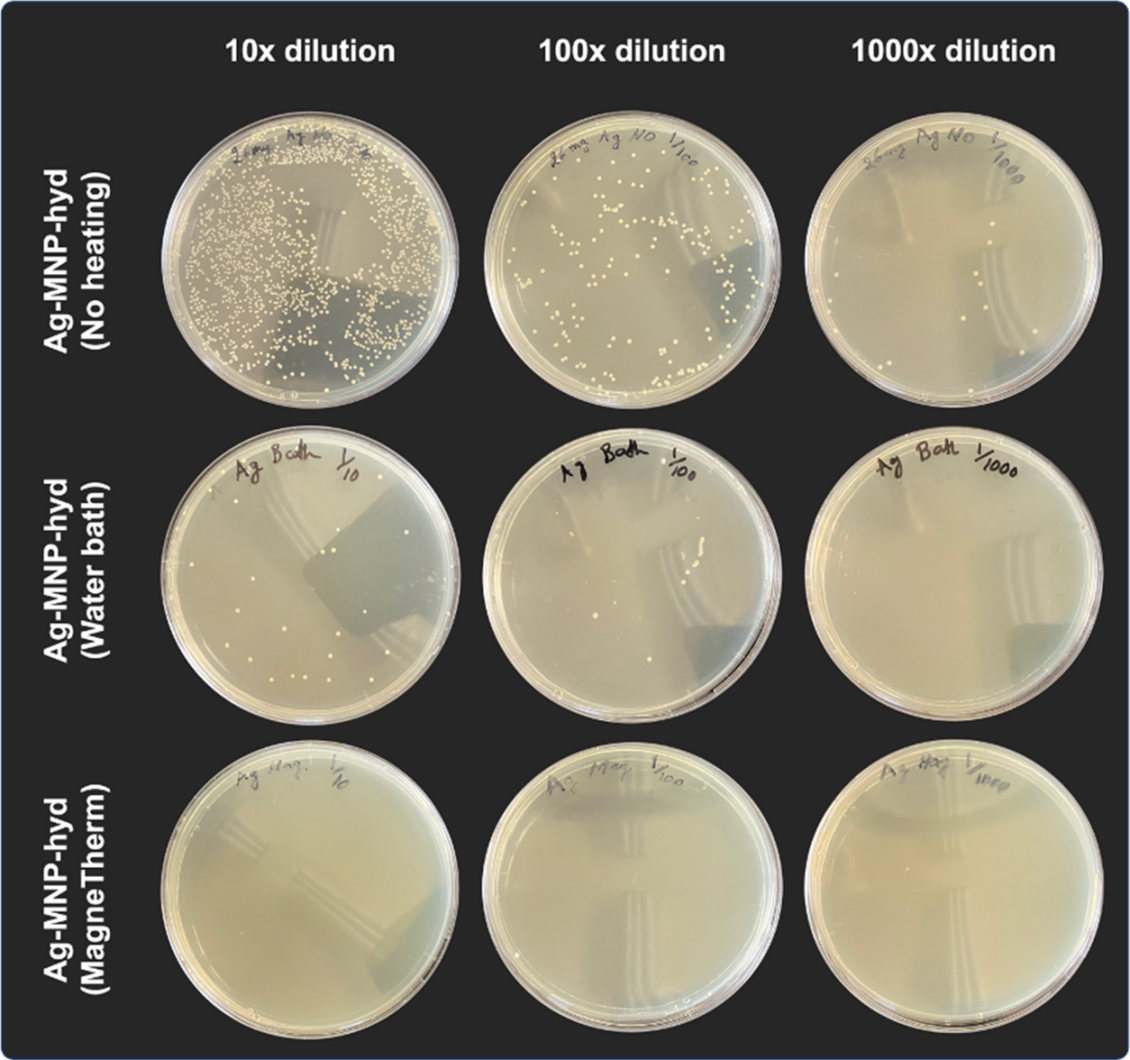
Plates of bacterial growth after serial dilutions at 10×, 100×, and 1000× under different conditions are shown.

**Figure 6:**
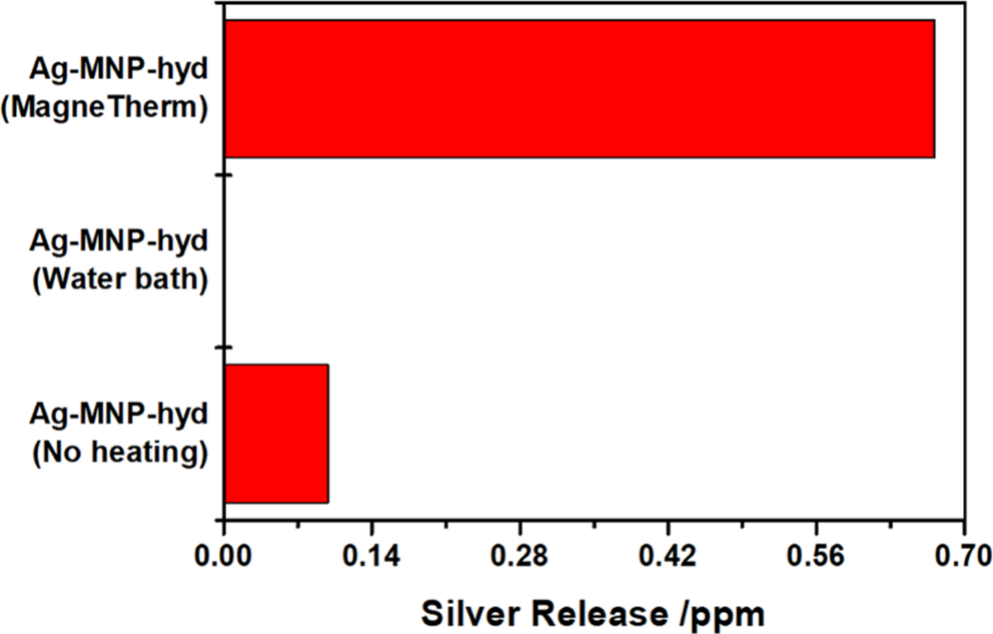
Atomic Absorption Spectroscopy (AAS) results showing silver concentration release (ppm) of Ag-MNP-hyd treated under different heating conditions for 15 min.

To test the dual antibacterial activity of postmetalated composites, three vials of bacterial *E. coli* broth were prepared with 40 mg/mL of Ag-MNP-Hyd in each vial and under similar silver concentration. Two vials were heated to 45 °C by using two different methods. One was heated by a water bath, and the other was heated by induction using the magnetherm, each for 15 min. With no heating, our Ag-MNP-hyd composites exhibited a bacterial inhibition of 17% compared to the positive control (Figures 4 and S8), which could be mainly due to the action of silver as an antibacterial agent. Serial dilution of this sample revealed a final bacterial concentration of 28,000 CFU/mL (Figure 5). AAS was conducted to check for silver release and give further insight into the mode of bacterial inhibition (Figure 6). As a result, 17% bacterial inhibition correlated to release of 9.83 × 10^–2^ μg/mL of silver (Figure 6). When our particles were subjected to heating by a water bath until 45 °C, bacterial inhibition increased to 45% when compared to the positive control (Figures 5 and S2). We believe that this increase in bacterial inhibition is due to the synergistic effect of heating, which was discussed above, that renders bacteria more vulnerable, combined with the antibacterial activity of silver nanoparticles. The effectiveness of our Ag-MNP-hyd microparticles is also evident when comparing them to bacterial growth plates for hyd and MNP-hyd of similar concentrations (Figure S9). This is evident when serial dilutions of the obtained sample revealed a final bacterial concentration of 2400 CFU/mL (Figure 5). Moreover, AAS of Ag-MNP-hyd heated by a water bath to similar temperatures showed no silver released (at least not above the detection limit of the flame-AAS) compared to the control (Figure 6).

Because the lack of silver release did not seem to affect the microparticles’ antimicrobial ability, we suspect that the mode of action of the latter is at least partly through direct contact with the bacterial species. A similar mode of action was observed with another work our lab did with another silver-doped material.^23^ Our group has shown that it is possible to elicit antibacterial inhibition by way of contact inhibition with nanocomposite membranes in which their surfaces were postmetalated with silver.^23^ Thus, we hypothesize that a similar mechanism is at play when it comes to Ag-MNP-hyd heated by a water bath. Direct contact exposure to silver NPs on the surface of the microparticles combined with external heating by the water bath probably disrupts bacterial biofilm formation, leading to an enhancement in overall antibacterial activity.

When *E. coli* was exposed to Ag-MNP-hyd heated by magnetic hyperthermia to 45 °C, the antibacterial activity increased to 81%, almost twice that of the same microparticles heated in a water bath (Figures 4 and S8). Serial dilutions of the sample reveal no bacterial growth, revealing that our composites achieved MBC at 40 mg/mL when exposed to heating by magnetic hyperthermia (Figures 4 and 5). Compared to the water bath-heated sample, which showed no silver release, AAS of the composites heated by induction revealed enhanced silver release with a concentration of 0.671 μg/mL compared to the control (0.0983 μg/mL) (Figures 6 and S10). Consequently, we believe that this increased silver, combined with the mechanical and thermal effects of MNPs on bacteria and biofilm disruption, mainly explain the synergy-enhanced antibacterial activity exhibited by the composites heated by magnetic hyperthermia compared to those heated by water bath. Furthermore, we hypothesize that the mechanical agitation produced by the oscillation of MNPs on the polymer matrix aided in the enhanced silver release observed. According to Li et al., poly-NIPAM composites collapse in size and shrink when exposed to temperatures that surpass the lowest critical solution temperature (LCST) of 35 °C.^62^ When heating our composites using a water bath to temperatures which greatly surpass the LCST of poly-NIPAM, we expect the homogeneous overall heating effect of the solution to shrink our hydrogels, potentially inhibiting silver release. Research done by Kamachi et al. even shows that silver release decreases when Ag-NIPAM nanocomposites with mesoporous silica are heated as the matrix physically shrinks.^63^ This is evident from Figures 6 and S10 when comparing the concentration of silver released by water bath-heated composites to the control (no heating). However, composites exposed to magnetic hyperthermia exhibited enhanced silver release and, thus, enhanced bacterial activity.

We believe that this difference in silver release could be attributed to the vibration/oscillation of MNPs in response to the alternating magnetic field of the magnetherm. Thus, enhanced silver release in this case could be mediated by the mechanical movement of MNPs within the hydrogel matrix and releasing silver despite the poly-NIPAM’s collapse. Alternatively, because of the difference in heating where magnetic hyperthermia is more heterogeneous and localized compared to the homogeneous and spread-out heating of the water bath, we believe that silver release, and consequently the dominating mode of action of the Ag-MNP-hyd composites, could be affected by the mechanism of hydrogel collapse. Without any AMF present, we believe that the main mode of action of silver, albeit less effective, is direct contact. However, with AMF, the mode of action mainly becomes diffusion of released silver into the solution. Our results demonstrate that our Ag-MNP-hyd composites exhibit synergistic dual antibacterial activity, enabled by the localized heating and mechanical abrasion of MNPs, as well as the antibacterial nature of silver. Figure S11 clearly shows the synergistic effect of heating by magnetherm combined with the postmetalation of silver such that having both aspects is necessary to achieve proper synergy and thus enhancement of antibacterial activity. Our results highlight the effectiveness of Ag-MNP-hyd microparticles in achieving MBC at an impressively low silver concentration of 0.671 μg/mL through magnetic hyperthermic exposure. This represents a significant improvement to our past work with different nanocomposites materials where MBC was at achieved at 95 and 6.5 μg/mL, demonstrating a 140-fold and 10-fold decrease in silver concentration, respectively.^21,23^ The synergistic hyperthermic and silver bactericidal properties of Ag-MNP-hyd are evident in the substantial inhibition of bacterial growth, even at very low silver concentrations. This shows that our microparticles exhibit a slow and controlled silver release, minimizing the potential for any silver toxicity.

## Conclusions

In conclusion, the incorporation of AgNPs into MNP-Hyd resulted in Ag-MNP-Hyd microparticles that were responsible for significant bacterial inhibition. This inhibition increased from 45 to 81% after our sample was heated using hyperthermic induction rather than heated in a water bath at 45 °C. The increase is due to the inductive heating effect of Ag-MNP-hyd, which is crucial to releasing silver nanoparticles to kill bacteria. We believe that when exposing Ag-MNP-hyd microparticles to heating by magnetic hyperthermia, the localized heating effect leading to bacterial biofilm disruption combined with the enhanced silver release makes bacteria more susceptible to death by the antimicrobial activity of silver, thus leading to better inhibition overall. These characteristics of magnetic hyperthermia amplify our microparticles’ antibacterial efficacy compared to heating by a water bath. The antibacterial efficacy of the postmetalated microparticles reached up to 81% achieving MBC at a very low silver concentration of 0.671 μg/mL when combined with magnetic hyperthermia. Overall, our findings suggest Ag-MNP-hyd composites can be used to serve as effective antibacterial materials, which could potentially be incorporated into sterilization systems such as water filtration. By integrating our microparticles into these systems, effective bacterial sterilization could be achieved with a very minute amount of silver release, limiting the potential for toxicity. This could be followed by the retrieval of our microparticles through a magnet, underscoring their versatility in different sterilization systems.

## Supporting Information Available

Detailed experimental data on the characterization of the prepared composites (PDF)
