# Supplementary material for: Synergistic Dual Antibacterial Activity of Magnetite Hydrogels Doped with Silver
Source: Langmuir. 2024 Oct 17;40(43):22865–74. doi: 10.1021/acs.langmuir.4c02964 (PMC11526350; doi:10.1021/acs.langmuir.4c02964)
Supplement: Supplementary file 1 — la4c02964_si_001.pdf [file la4c02964_si_001.pdf]

SUPPORTING INFORMATION

The Synergistic Dual Anti-Bacterial Activity of Magnetite Hydrogels Doped with Silver

Mohamad Wehbe, Rayan Kadah El Habbal, Jad Kaj and Pierre Karam*

Chemistry Department, American University of Beirut, P.O.Box 11-0236, Riad El-Solh, 1107 2020 Beirut, Lebanon

*Pierre Karam: pierre.karam@aub.edu.lb

DLS Measurement Curves:

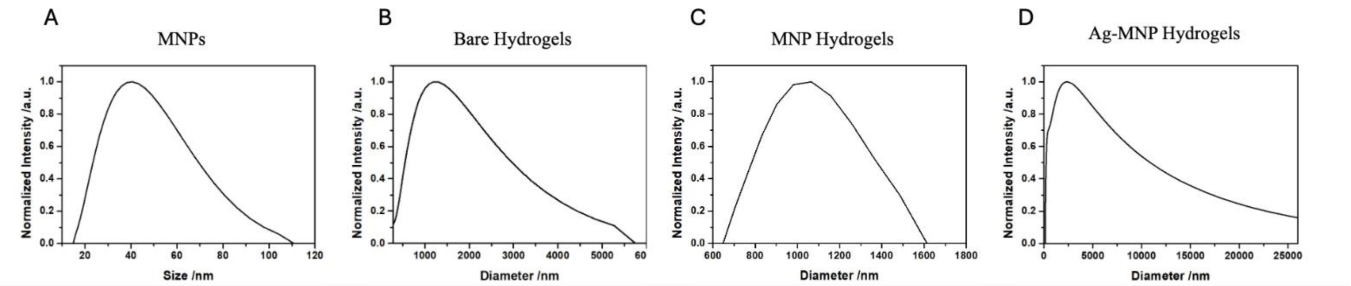

Figure S1. Size distribution of different synthesized particles acquired through DLS

XRD Analysis:

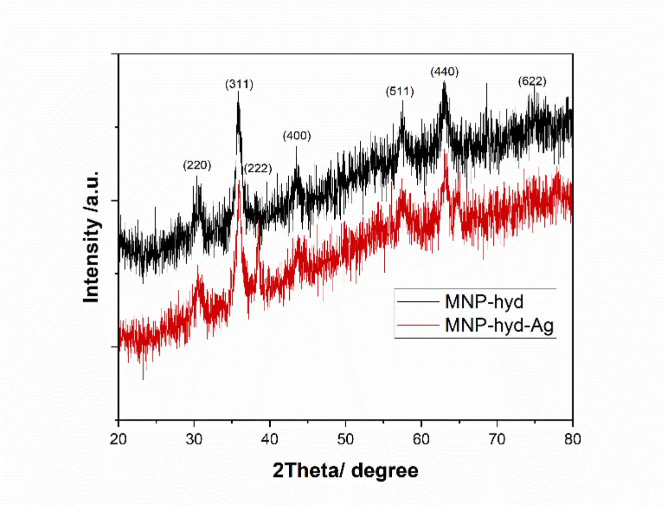

Figure S2. XRD patterns of MNP-hyd, and MNP-hyd-Ag microparticles

SEM Images:

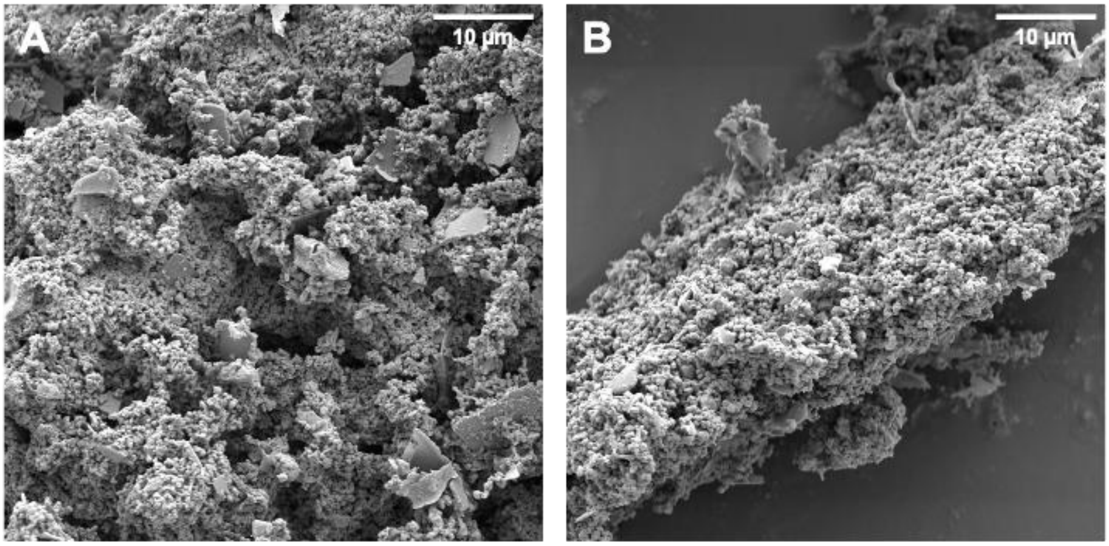

Figure S3. SEM of MNP-hyd doped with 180 mg of silver nitrate

Heat Measurements of Ag-MNP-hyd at different $AgNO_3$ concentrations:

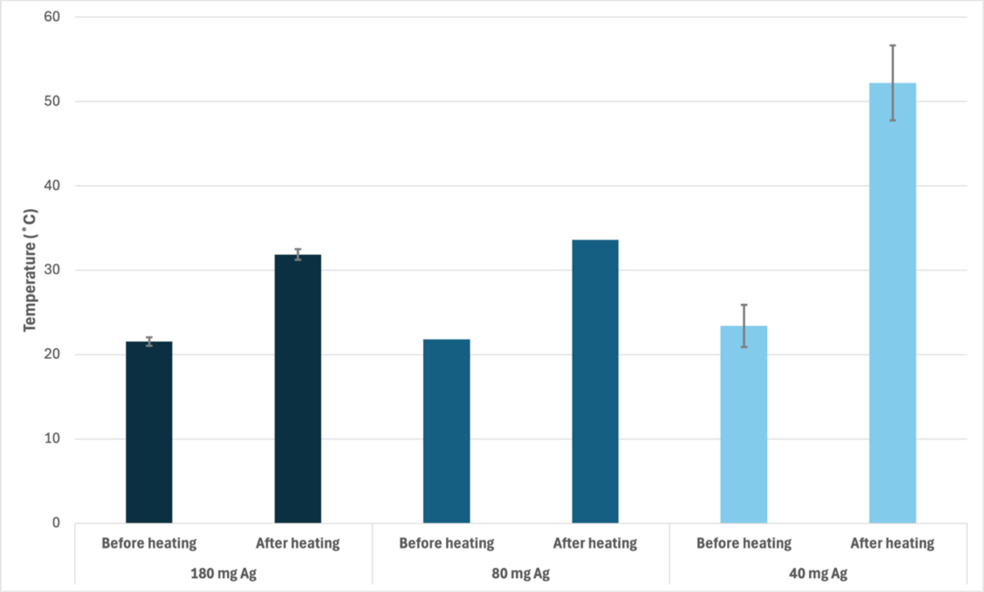

Figure S4. Temperature of broth with Ag-MNP-hyd before and after inductive heating for 15 mins at different $AgNO_3$ weights used

EDX Measurements:

EDX was used to qualitatively visualize and detect silver nanoparticles on the surface of our Ag-MNP-hyd microparticles, confirming the presence and embedment of silver into the polymeric matrix.

**Sample**	**Percent by Weight (%)**				
	Carbon	Nitrogen	Oxygen	Iron	Silver
Bare MNP	6.23 ± 2.21	0	29.53 ± 2.41	64.25 ± 0.21	0
Bare hyd	71.08 ± 0.51	15.12 ± 0.17	13.80 ± 0.68	0	0
MNP-hyd	12.90 ± 4.47	0	32.12 ± 0.092	54.99 ± 4.57	0
Ag-MNP-hyd	12.84 ± 6.00	1.01 ± 1.75	31.8 ± 4.14	51.46 ± 4.36	3.47 ± 2.31

Figure S5. EDX data showing the composition of different synthesized complexes in percentage by weight (%).

Heating Measurements of Microparticles before and after Induction:

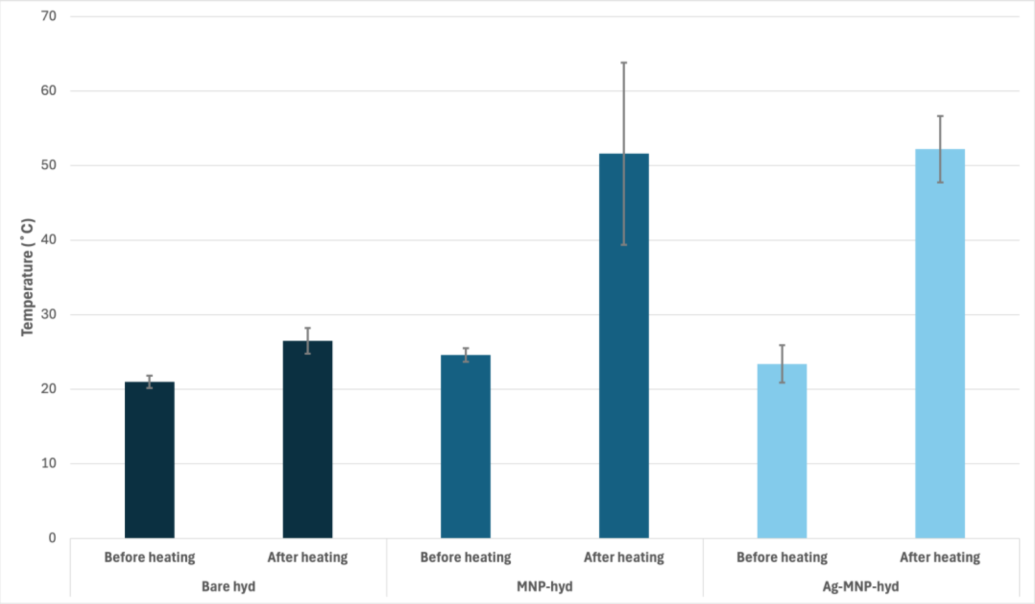

Figure S6. Temperature of the solution (bacteria in broth + material) before and right after inductive heating for 15 mins. Error bars represent the standard deviation of 5 samples

Normalized Bacterial Inhibition:

This figure shows the normalized bacterial inhibition % of MNP-hyd under different heating conditions. MNP-hyd with no heating showed minimal inhibition compared to MNP-hyd heated by either water bath or magnetherm. MNP-hyd heating induction through magnetherm showed enhanced antibacterial activity when compared to the same particles heating by water bath.

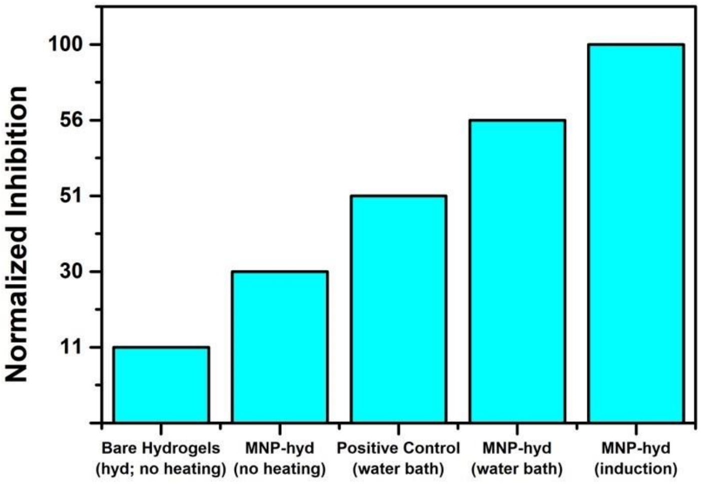

Figure S7. The relationship between normalized bacterial inhibition and different non-metalated nanocomposites (25-40 mg/mL).

The below figure shows the normalized antibacterial inhibition of Ag-MNP-hyd microparticles under different heating conditions. Ag-MNP-hyd heated by magnetic hyperthermia showed greater antibacterial activity when compared to the same particles under water bath heating conditions and particles without heating at all.

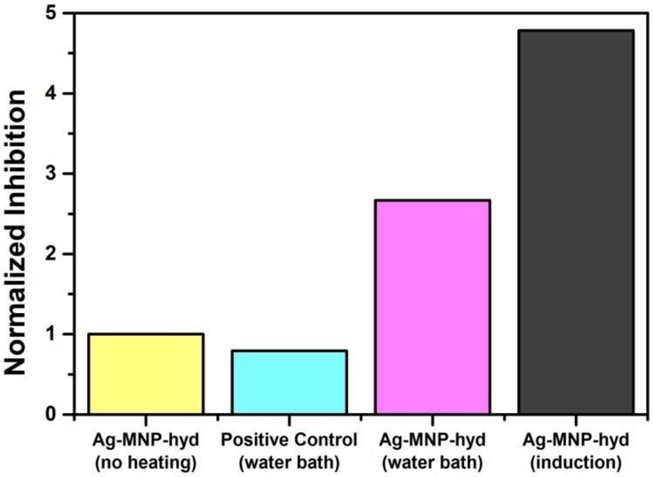

Figure S8. Relationship between normalized bacterial inhibition and post-metalated nanocomposites (40 mg/mL) under different heating conditions

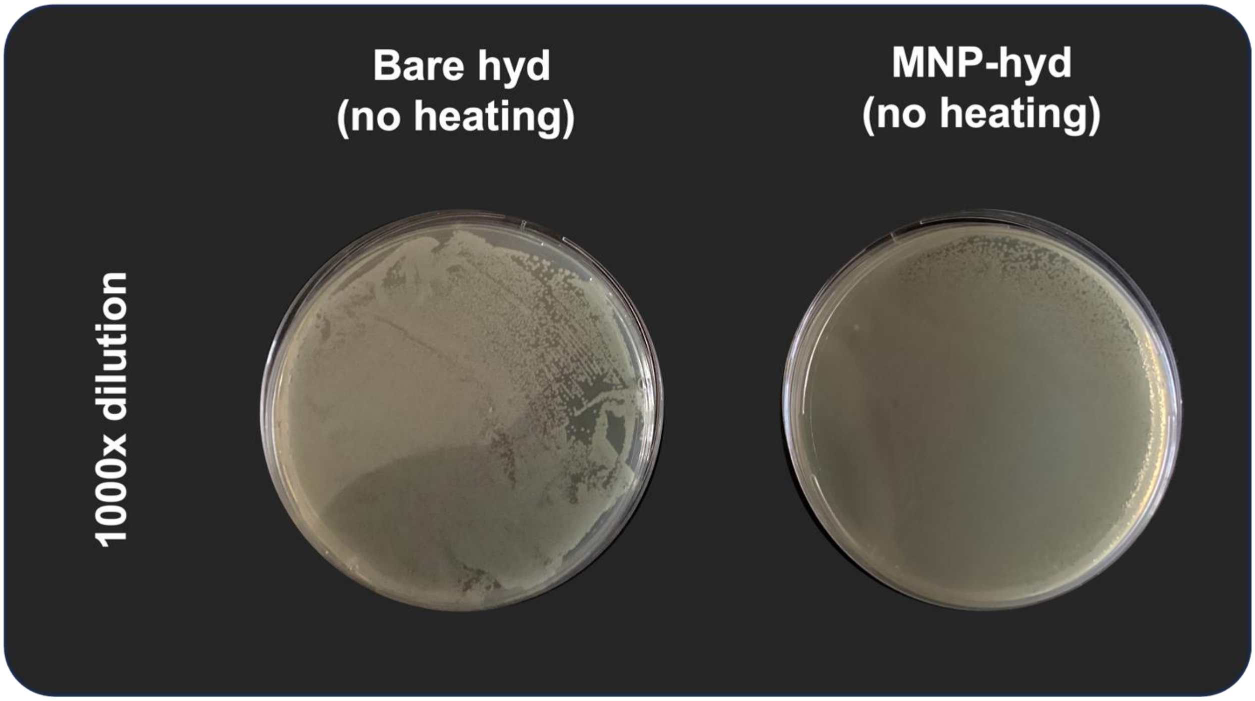

Figure S9. Plates of bacterial growth after 1000X serial dilution of for bare hyd and MNP-hyd

Silver Release:

This figure shows the normalized silver release concentration of Ag-MNP-hyd microparticles under different heating conditions. Without any heating, silver release is minimal. When heating by water bath, silver release decreased to zero. On the other hand, heating by magnetic hypethermia led to enhanced silver release from our microparticles. The mechanism of release is further explored in the main manuscript.

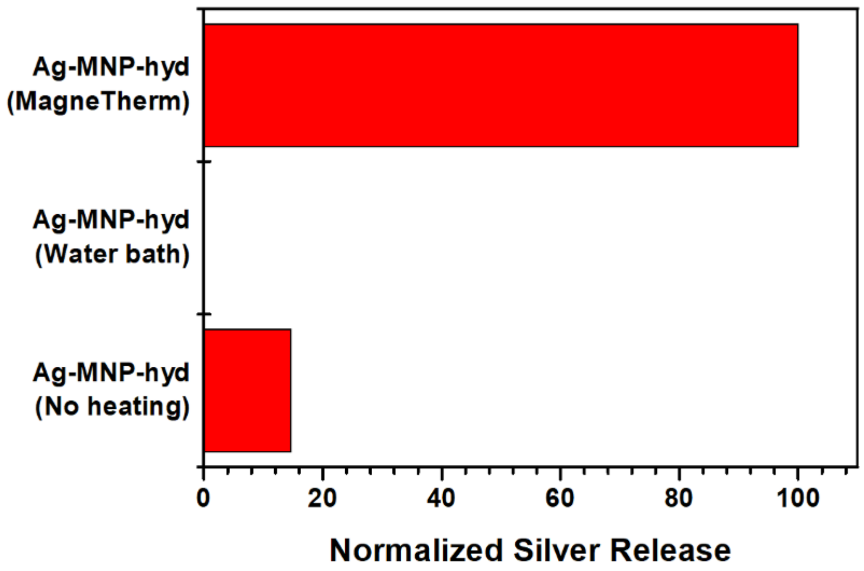

Figure S10. Atomic Absorption Spectroscopy (AAS) results showing the normalized silver concentration release of Ag-MNP-hyd treated under different heating conditions for 15 minutes

Percentage Inhibition:

This figure shows the percentage inhibition of MNP-hyd compared to Ag-MNP-Hyd under different heating conditions. Ag-MNP-hyd exhibited better antibacterial activity overall under all conditions. Moreover, this figure shows increasing antibacterial activity when going from no heating conditions to water bath to induction.

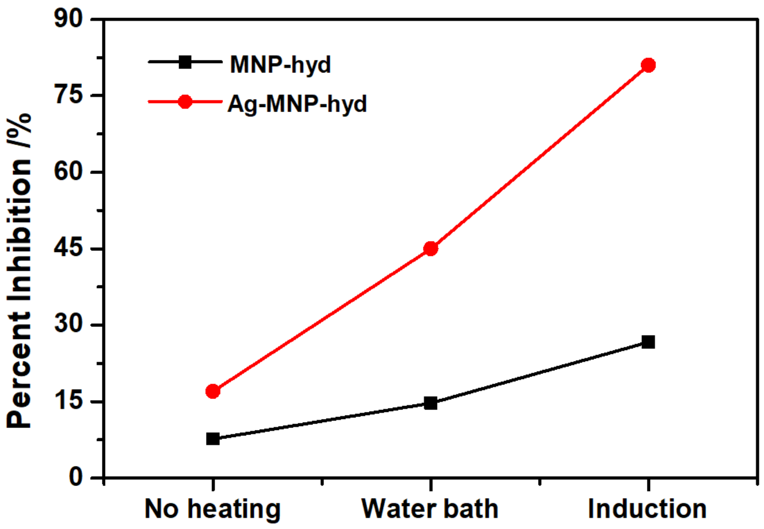

Figure S11. Relationship between bacterial inhibition (%) and MNP-hyd and Ag-MNP-hyd under different heating conditions

Atomic Absorption Spectroscopy:

This figure shows the calibration curve used to calculate the silver concentration released from Ag-MNP-hyd under different heating conditions analyzed by AAS.

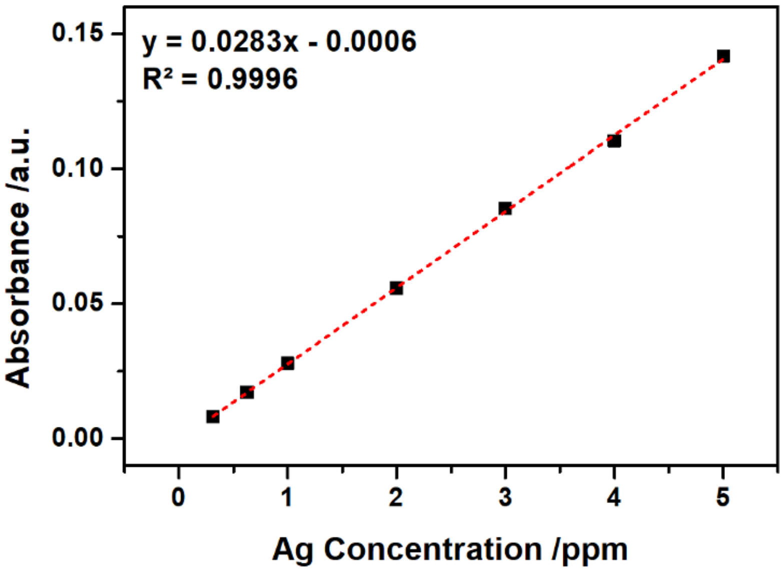

Figure S12. Calibration curve showing the relationship between absorbance (a.u.) and Ag concentration (ppm)

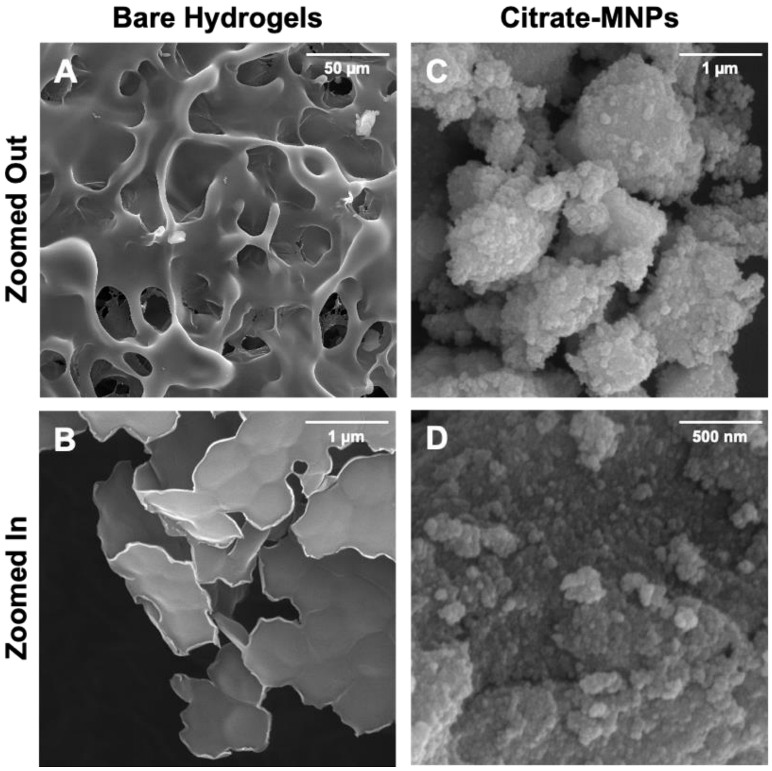

Figure S13. SEM images of bare hydrogels and citrate-MNPs.
